# ﻿*Impatiensbijieensis* (Balsaminaceae), a new species from karst plateau in Guizhou, China

**DOI:** 10.3897/phytokeys.192.77517

**Published:** 2022-03-04

**Authors:** Liu-Yi Ren, Yi Chen, Tao-Hua Yuan, Rong-Xin Huang, Mei-Jun Li, Xin-Xiang Bai

**Affiliations:** 1 College of Forestry, Guizhou University CN-550025 Guiyang, China Guizhou University Guiyang China

**Keywords:** Balsaminaceae, China, *Impatiens*, morphology, new species

## Abstract

*Impatiensbijieensis* X.X. Bai & L.Y. Ren, **sp. nov.** from northwest Guizhou Province, China, is described and illustrated. This new species is distributed discontinuously in Jiulongshan, Dafang County and Dajiucaiping, Hezhang County, both of which are in the Wumeng Mountain area, a karst plateau landform. The new species is morphologically similar to *I.pterosepala* Hook.f., *I.lasiophyton* Hook.f. and *I.leptocaulon* Hook.f. in height and flower shape and it especially resembles *I.lasiophyton* in pilosity. However, it differs in its deep purplish-red flower, 2-lobed lower sepal apex and cylindrical capsule. A detailed description, colour photographs and a provisional IUCN Red List assessment are provided along with discussions of its geographical distribution, ecology and morphological relationships with other similar species.

## ﻿Introduction

The genus *Impatiens*Linnaeus (1753: 937), belonging to the Balsaminaceae, consists of more than 1000 species, mainly distributed in the montane forests of the tropics and subtropics of the Old World with five centres of diversity, namely tropical Africa, Madagascar, south India and Sri Lanka, eastern Himalaya and Southeast Asia (Grey-Wilson 1980; Song et al. 2003; Yuan et al. 2004; Mabberley 2017). In recent years, a few new species have also been found in the northern temperate regions of Europe, Russia and China, as well as North America (Liao et al. 2021). Currently, there are more than 349 species of *Impatiens* in China (Yuan et al., in press) which are distributed mainly in the southwest and northwest mountainous regions, especially in southwest Provinces (including Guizhou, Yunnan, Sichuan) (Chen 2001; Chen et al. 2007; Cai et al. 2015; Kuang 2015; Tan et al. 2015; Ding et al. 2016, 2017; Xia et al. 2019; Gu et al. 2021; Liao et al. 2021; Peng et al. 2021b; Song et al. 2021a, b, c) and 61 species of *Impatiens* have been reported in Guizhou Province (Xiong and Yang 2009; Cong 2010; Kuang et al. 2014; Luo and Deng 2015; Peng et al. 2021a; Yu et al. 2021; Yuan et al. in press).

*Impatiens* are morphologically characterised by their petals always united in pairs into lateral, united petals; fruit a fleshy, explosive capsule; seeds often dispersed elastically from valves when ripe (Chen et al. 2007). From September 2014 to October 2021, during our field investigation in Bijie City, northwest Guizhou Province, China, we encountered an unknown *Impatiens* species. The plants were found growing in gullies between gently sloping mountain meadows of karst plateau, this special habitat distinguishing our plants from other known species. After a thorough morphological study, based on literature (Xiong and Luo 1989; Chen 2001; Chen et al. 2007; Yu 2012) and herbarium material (GZAC!), we concluded that this *Impatiens* species should be placed in I.subg.Impatiens as it differed from previously reported or described taxa and we describe it here as a new species.

## ﻿Materials and methods

The material for this study was mainly collected from the survey of wild ornamental plant resources in Guizhou Province, China. The morphological description of the new species was based on careful examination of fresh material in the field and herbarium specimens. Comparisons with other species were made to virtual herbarium specimens (GZAC, HC, HIB, IBK, IBSC and PE), photographs and literature (Hooker 1908a; Grey-Wilson 1980; Chen 2001; Chen et al. 2007; Yu 2012).

## ﻿Taxonomic treatment

### ﻿ Impatiens bijieensis

Taxon classificationPlantae

X.X. Bai & L.Y. Ren sp. nov.

F382138C-8113-58F2-A739-3C97C68EF6B3

urn:lsid:ipni.org:names:77265014-1

Figs 1 , 2 , 3A–C

#### Type.

China. Guizhou: Hezhang County, Xingfa Town, Dajiucaiping, 2763 m alt., 29°59'53"N, 104°45'29"E, 20 Aug 2021, *X.X. Bai & L.Y. Ren DJCP 20210820* (holotype: GZAC!; isotype: PE!).

**Figure 1.**
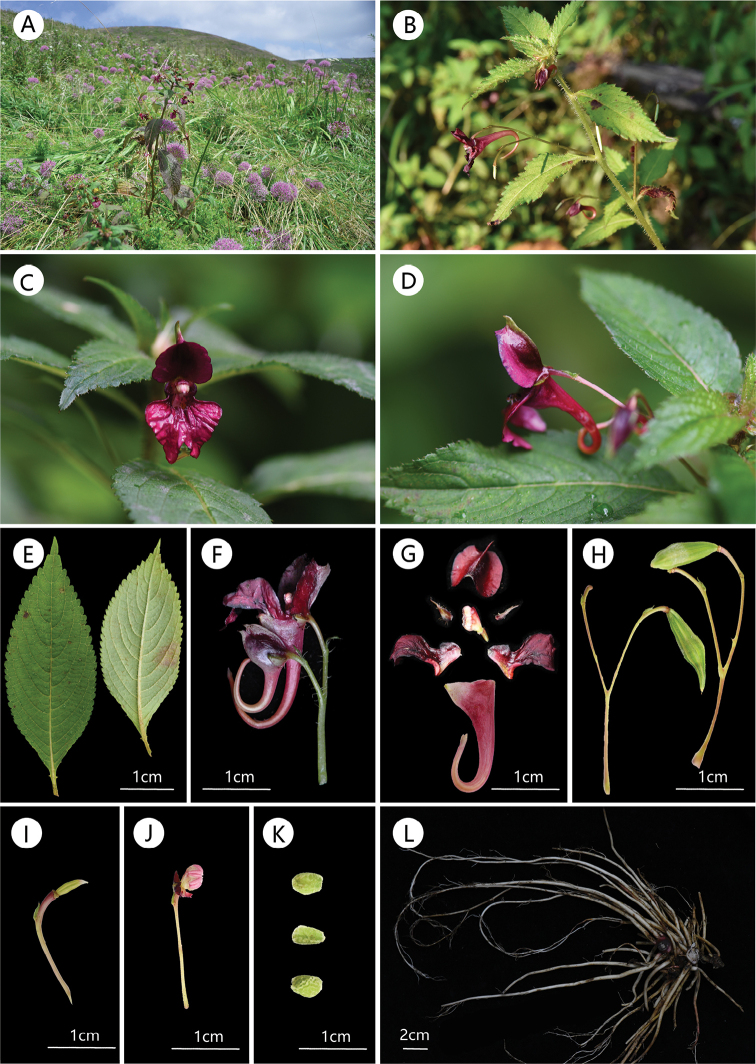
*Impatiensbijieensis***A** habit **B** plant **C** flower in face view **D** flower in lateral view **E** leaf adaxial surface (left) and abaxial surface (right) **F** inflorescence **G** flower dissected **H** fruit **I** ovary **J** anther **K** seeds **L** root. Photos by Xin-Xiang Bai.

#### Diagnosis.

*Impatiensbijieensis* X.X. Bai & L.Y. Ren, sp. nov. is similar to *I.pterosepala*, *I.lasiophyton* and *I.leptocaulon* in plant height, leaf blade shape and flower shape, especially resembling *I.lasiophyton* in its pilosity and obtuse anther apices, but distinguished by its deep purplish-red flower, linear-lanceolate bract, apex 2-lobed lower sepal and cylindrical capsule.

**Figure 2.**
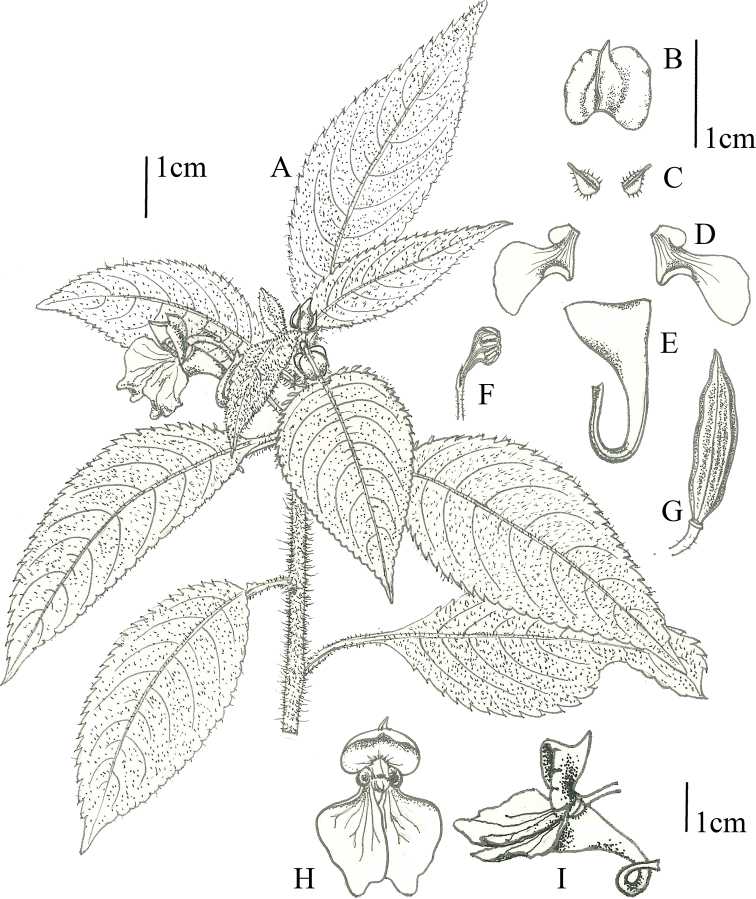
*Impatiensbijieensis***A** habit **B** dorsal petal **C** lateral sepal **D** lateral united petal **E** lower sepal **F** anther **G** fruit **H** flower in face view **I** flower in lateral view. Drawings by Yi Chen, based on holotype specimen.

#### Description.

Perennial herb, 30–60 cm high, densely pilose. Root-system developed, with adventitious roots on lower stem nodes. Stem fleshy, erect, branched. Leaves simple, alternate, aggregated at stem apex; petiole 0.3–0.8 cm long; leaf blade elliptic, ovate or ovate-lanceolate, 3.5–5 cm × 1.3–1.8 cm, base cuneate, with a pair of conical glands, margin serrulate, apex acuminate, adaxial surface densely pilose, green; abaxial surface pilose along veins, pale green, lateral veins 6–8 pairs. Inflorescences axillary, 2- or 3-flowered. Peduncles 1.5–1.8 cm long, pedicels 0.4–0.8 cm long, slender, bracteate above middle; bract 1, persistent, linear-lanceolate, 0.3–0.5 cm long, abaxial mid-vein thickened, margin membranous, ciliate.

Flowers deep purplish-red, 2.3–2.5 cm long. Lateral sepals 2, ovate, ca. 0.5 cm × 0.2 cm, margin ciliate, mid-vein carinate, apex acuminate or caudate. Lower sepal navicular, ca. 0.5 cm deep (excluding spur), mouth vertical, ca. 0.8 cm wide, narrowed into an incurved, long spur, ca. 2 cm, apex 2-lobed. Dorsal petal reniform, ca. 0.8 cm × 1.5 cm, base truncate or suborbicular, apex concave, abaxial mid-vein carinate, apex acuminate. Lateral united petals sessile, ca. 1.7 cm long, deep purplish-red with whitish-pink base and reddish patches near auricle, 2-lobed; basal lobes ca. 0.4 cm × 0.2 cm, oblong to obovate-oblong; distal lobes ca. 1 cm × 0.6 cm, dolabriform. Stamens 5, filaments linear, anther apices obtuse; ovary 5-carpellate, narrowly fusiform, 0.4–0.5 cm, erect. Capsule fusiform, 1.3–1.8 cm long, apex acuminate, 5-valved, fleshy. Seed ellipsoid, surface with irregular protrusions.

#### Etymology.

The specific epithet ‘bijieensis’ refers to the type locality where the new species was found, located in Bijie City, northwest Guizhou Province, China. The Chinese name is given as “毕节凤仙花”.

#### Phenology.

Flowers and fruits from August to October.

#### Distribution.

*Impatiensbijieensis* has been observed in Bijie City, Guizhou Province with subpopulations in Hezhang County and Dafang County. We found at Dajiucaiping, Hezhang that the plants grow along the creek on the slopes of gentle hilltops or on the side slope of the ridge, while some were found below the cliffs where water was dripping from streams. At Jiulongshan, Dafang, the population was distributed in evergreen broad-leaved forest margins.

#### Ecology.

*Impatiensbijieensis* was collected at elevations of 1915–2800 m. Species of *Rubuscoreanus* Miq. (Rosaceae), *Elatostemainvolucratum* Franch. et Sav. (Urticaceae) and *Alliumwallichii* Kunth (Liliaceae) were found to grow in the vicinity of this species.

#### Conservation status.

This species is currently known only from Bijie City, Guizhou Province, China with two subpopulations. The Extent of Occurrence (EOO) is less than 100 km^2^ and the known Area of Occupancy (AOO) is less than 15 km^2^. The conservation status can be evaluated as Vulnerable (VU) D2, based on the IUCN Red List Categories and Criteria (IUCN 2019). About 150 and 300 individuals were known in the two subpopulations of Hezhang County and Dafang County, both of which are exposed to human disturbance.

#### Additional specimen examined.

**China. Guizhou**: Bijie City: Dafang County, Jiulongshan, 27°19'37"N, 105°52'50"E, 1915 m alt., 8 Sep 2021, *X.X. Bai et al.*, *JLS 20210908* (GZAC!).

## ﻿Discussion

*Impatiensbijieensis* is similar to *I.pterosepala* in floral morphology; both have dolabriform distal lobes of lateral united petals and ovate lateral sepals. The former differs from the latter in being pilose (vs. glabrate), having 2–3-flowered (vs. 1-flowered) inflorescences, deep purplish-red (vs. pale purple or purple-red) flower colour, serrulate (vs. crenate) leaf blade margin, conical (vs. globose) glands at leaf bases, mid-veins of dorsal petals carinate (vs. thickened, entire or undulate), margin of lateral sepals ciliate (vs. sometimes denticulate at one side), abaxial mid-veins of lateral sepals carinate (vs. narrowly carinate), anther apices obtuse (vs. acute) and capsules cylindrical (vs. linear).

*Impatiensbijieensis* is similar to *I.lasiophyton* mostly in its pilosity and elliptic, ovate or ovate-lanceolate leaf blades, but it can be easily distinguished from the latter by its deep purplish-red (vs. yellow or white) flowers, thickened abaxial mid-vein and ciliate margin, membranous bract (vs. hirsute bract and inconspicuous mid-vein), navicular and apically 2-lobed lower sepal (vs. broadly funnelform and unlobed), apex concave, abaxial mid-vein carinate dorsal petal (vs. apex obtuse, abaxial mid-vein thickened, cristate apically), margin of lateral sepal ciliate (vs. lateral sepal hirsute), and cylindrical (vs. linear) capsules.

**Figure 3.**
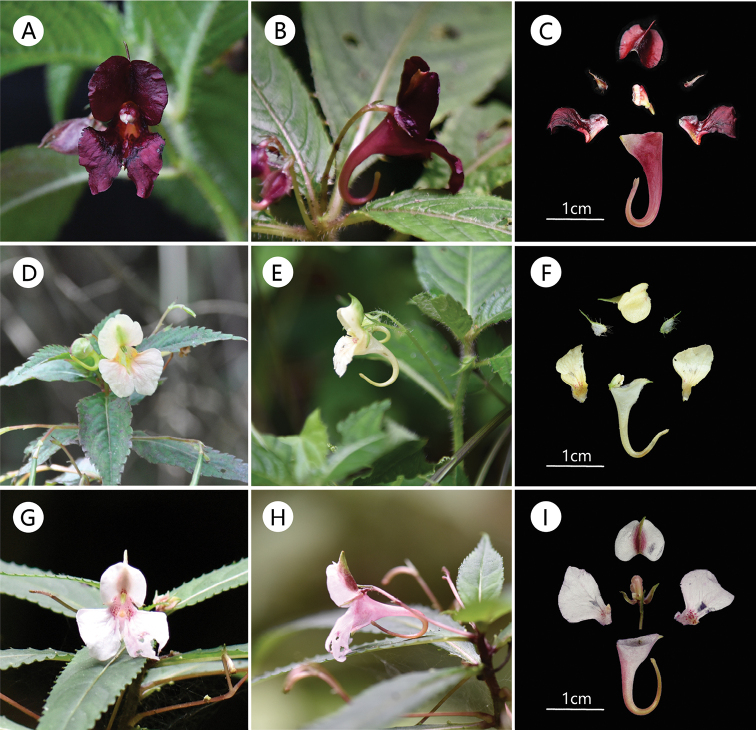
*Impatiensbijieensis* (**A–C**) **A** flower in face view **B** flower in lateral view **C** flower dissected (Photos by X.X. Bai, in Hezhang County, 20 August 2021). *Impatienslasiophyton* (**D–F**) **D** flower in face view **E** flower in lateral view **F** flower dissected (Photos by X.X. Bai, in Duyun City, 8 July 2021). *Impatiensleptocaulon* (**G–I**) **G** flower in face view **H** flower in lateral view **I** flower dissected (Photos by X.X. Bai, in Guiyang City, 22 June 2019).

*Impatiensbijieensis* is also similar to *I.leptocaulon* in the length of its petiole, serrulate leaf blade margin and navicular lower sepal, but differs from its adaxial surface densely pilose and abaxial surface pilose along veins (vs. glabrous), ciliate margined lateral sepals and carinate mid-vein (vs. hyaline margined, denticulate on one side). In order to illustrate the morphological circumscription of this new species, we compare the new species with three species with similar morphological characters in Table 1: *Impatienspterosepala* Hook.f. (1910:274), *I.lasiophyton* Hook.f. (1908b: t. 2871) and *I.leptocaulon* Hook.f. (1908c: t. 2872). Colour photographs of *I.bijieensis*, *I.lasiophyton* and *I.leptocaulon* are given in Fig. 3 and all of them were taken in Guizhou Province.

**Table 1.** Comparison of morphological characters in *Impatiensbijieensis*, *I.pterosepala* (data from Hooker 1910), *I.lasiophyton* (data from Hooker 1908b) and *I.leptocaulon* (data from Hooker 1908c).

Character	*I.bijieensis*	*I.pterosepala*	*I.lasiophyton*	*I.leptocaulon*
Length of petiole	0.3–0.8 cm	1.5–2.0 cm	1–3 cm	0.5–1.5 cm
Leaf blade	with a pair of conical glands at base, base cuneate, margin serrulate, adaxial surface densely pilose, abaxial surface pilose along veins	with 2 globose basal glands, base cuneate, margin crenate, both surfaces glabrous	base acute, margin coarsely crenate or crenate-serrate, both surfaces hirsute	with few basal glands, base narrowly cuneate, margin serrulate, both surfaces glabrous
Bract	bracteate above middle, linear-lanceolate	bract above middle; lanceolate	bracteate below flower; lanceolate	bracteate above middle; lanceolate
Flower	deep purplish-red	pale purple or purple-red	yellow or white	purple-red
Lateral sepal	2, ovate, margin ciliate, mid-vein carinate, apex acuminate or caudate	2, ovate, margin sometimes denticulate at one side, abaxial mid-vein narrowly carinate, apex acuminate	2 (or 4), subovate, hirsute, apex cuspidate	2, subovate, long cuspidate, inequilateral, hyaline margined, denticulate on one side
Lower sepal	navicular, narrowed into an incurved, long spur, spur apex 2-lobed	narrowly funnelform, narrowed into an incurved, slender spur	broadly funnelform, gradually narrowed into an incurved spur	navicular, narrowed into an incurved, long spur
Dorsal petal	reniform, base truncate or suborbicular, apex concave, abaxial mid-vein carinate	orbicular, base cordate, apex slightly emarginate, shortly rostellate, abaxial mid-vein thickened, entire or undulate	orbicular, base cordate, apex obtuse, abaxial mid-vein thickened, cristate apically	orbicular, base unknown, apex rostellate, abaxial mid-vein carinate
Basal lobes	oblong to obovate-oblong	oblong	small or rudimentary	orbicular, small
Distal lobes	dolabriform	broadly dolabriform, larger	broadly dolabriform or sublunar	obovate-oblong
Anther apex	obtuse	acute	obtuse	obtuse
Capsule	cylindrical	linear	linear	linear
Elevation	1915–2800 m	1500–1700 m	1700–2700 m	1200–2000 m

The morphological characters, including perennial habit, racemose inflorescence, 5-carpellate ovary, cylindrical capsule and ellipsoidal seeds, indicate that *Impatiensbijieensis* is a member of the I.subg.Impatiens. It is known that *Impatiens* mainly grow in places with high relative temperature and low elevations, yet this new species is found in a karst plateau area that is characterised by its cold climate and high altitude. These ecological characters distinguish it from morphologically-similar species. Additionally, because of the development of tourism, its habitat is currently threatened by human activities.

## Supplementary Material

XML Treatment for Impatiens bijieensis
